# On Certain Forms of Heart Disease, and Their Liability to Terminate in Sudden Death

**Published:** 1865-11

**Authors:** A. T. H. Waters

**Affiliations:** Liverpool


### On Certain Forms of Heart Disease, and their Liability to Terminate in Sudden Death.

BY A. T. H. WATERS, M. D.

The disease of the heart which is most liable to terminate in sudden death is, undoubtedly, fatty degeneration of its muscular fibre. It is unnecessary to refer to statistics to prove this statement; the experience of most practitioners will confirm it. When fatty disease attacks the muscular substance of the heart, there is a gradual obliteration of its contractile element, and a gradual diminution of its contractile power. To such an extent does this sometimes take place, that, on making an examination of the tissue of the heart, we find but little evidence of its muscular nature; and we are surprised, not that death has occurred, but that life has been so prolonged. Death not unfrequently takes place suddenly in this disease without the previous occurrence of any well-marked symptom, such as to arrest the attention of the patient and warn him of his dangerous malady; and although it is probable that, in all such cases, a careful examination would reveal evidence of enfeebled heart, or of certain reflex phenomena, slight, but important in a diagnostic point of view, yet the absence of prominent features—such, for instance, as usually characterize the progress of valvular disease of the heart and of certain affections of its muscular walls—often leads the sufferer to imagine that no serious malady exists. And it is a circumstance of no little interest and importance that even when fatty degeneration of the heart is far advanced we occasionally find the pulse moderately full. I have known instances where this condition of pulse has misled as to the nature of the disease.

But beyond this question of the great liability of fatty disease of the heart to terminate in sudden death, there are some further points in relation to sudden death from valvular disease of great practical importance. Among these are—1st, *the probabilities of sudden death in valvular disease;* and, 2d, *the particular form of valvular disease most liable to terminate in sudden death.*

In regard to the first point, I think it may be safely affirmed that, speaking generally, the proportion of cases of valvular disease terminating in sudden death is very small. In the large majority of cases death results slowly, from the secondary consequences of the affection, dropsy or some other diseased condition. Dr. Barclay has recorded a series of seventy-nine fatal cases of valvular disease which occurred in St. George's Hospital. Two only of the deaths are mentioned as having been sudden. This proportion of sudden deaths is very small, and perhaps must not be taken as the usual one. My own experience certainly gives a larger proportion.

In relation to the second point, the particular form of valvular disease most likely to terminate in sudden death, I am not aware that any statistical tables exist which would tend to determine the question. It would be a matter of great moment, considering the strong impression which prevails amongst the public of the great liability to sudden death in heart disease, if we could arrive at any precise conclusions in regard to this subject—if, in fact, we could by the examination of a large number of cases deduee a rule of probability applicable to these valvular diseases. The experience of a single individual, unless extraordinarily large, would scarcely serve for any definite conclusions; but if a number of practitioners were to direct their attention to this particular point, the most valuable statistics might be obtained. Dr. Walshe, in the last edition of his work on Diseases of the Heart, has entered somewhat into this question, and he states that, according to his experience, the form of valvular disease most liable to terminate in sudden death is uncomplicated aortic regurgitation. On the other hand, Dr. Stokes is of opinion that mitral regurgitant disease is most liable to fatal syncope.

Theoretical considerations lead me to the conclusion that, of the two forms of disease just mentioned, mitral regurgitation is more liable to terminate in sudden death than aortic regurgitation. In the latter affection the dilatation and hypertrophy of the left ventricle are especially salutary. If the disease is chronic, the ventricle gradually adapts itself to its altered requirements, and, for a time, but few symptoms sufficiently severe to attract the attention of the patient may result. In consequence of its dilatation and hypertrophy, the ventricle is able both to hold a larger quantity of blood than in health, and to contract with greater power, so as to throw all its contents into the aorta. The arterial tubes thus become well filled by each ventricular systole—in fact, they receive more blood than when the heart is in a normal condition; but in consequence of the imperfect closure of the semilunar valves they lose a portion of this blood, and hence the sudden collapse of the arteries after their diastole, which gives so peculiar a character to the pulse in aortic regurgitant disease. Now, as the structures of the body get well supplied with blood *so long as the ventricle retains its vigor*, there is, speaking comparatively, but little element of syncope and sudden death. On the other hand, when the mitral valve is diseased, so as to allow of regurgitation, a portion of the blood which ought to go to fill the arteries passes back into the left auricle. Hence these cases are characterized by a small pulse—a pulse of little volume. If the amount of regurgitation is large, the quantity of blood passing into the systemic vessels will necessarily be small. Here we have the element of syncope; and, as the result of an unusually feeble contraction of the ventricle, or some embarrassment to its action, sudden death may ensue.—Whether such embarrassment is more likely to take place in mitral than in aortic regurgitation is a subject for careful study and observation.

On looking over the cases of sudden death amongst my own patients, I find that I have had about an equal proportion of deaths from the two forms of disease to which I have referred, and consequently my own experience tends neither to confirm nor to contradict the opinion I have expressed on theoretical grounds. The question is one especially for statistics, and it is chiefly with the view of eliciting facts, and the opinions of those who have had a larger experience than I have, that I have brought the subject forward.

My own belief is, that it is a very rare thing for valvular disease to produce sudden death, unless the muscular substance of the heart has undergone a weakening, or a degeneration of its fibre. As long as the muscle retains its vigor, the grand cause of syncope is wanting. In all cases of sudden death from heart disease in which I have made a post-mortem examination, I have found fatty degeneration of the muscular fibre to a greater or less extent. The recognition of this fact is of great importance, as it points out the line of practice that should be adopted in the management of all cases of valvular disease.—*Lancet.*

Liverpool, September, 1865.

